# Wikipedia network analysis of cancer interactions and world influence

**DOI:** 10.1371/journal.pone.0222508

**Published:** 2019-09-19

**Authors:** Guillaume Rollin, José Lages, Dima L. Shepelyansky

**Affiliations:** 1 Institut UTINAM, CNRS, UMR 6213, OSU THETA, Université de Bourgogne Franche-Comté, Besançon, France; 2 Laboratoire de Physique Théorique, IRSAMC, Université de Toulouse, CNRS, UPS, Toulouse, France; USC Information Sciences Institute, UNITED STATES

## Abstract

We apply the Google matrix algorithms for analysis of interactions and influence of 37 cancer types, 203 cancer drugs and 195 world countries using the network of 5 416 537 English Wikipedia articles with all their directed hyperlinks. The PageRank algorithm provides a ranking of cancers which has 60% and 70% overlaps with the top 10 deadliest cancers extracted from World Health Organization GLOBOCAN 2018 and Global Burden of Diseases Study 2017, respectively. The recently developed reduced Google matrix algorithm gives networks of interactions between cancers, drugs and countries taking into account all direct and indirect links between these selected 435 entities. These reduced networks allow to obtain sensitivity of countries to specific cancers and drugs. The strongest links between cancers and drugs are in good agreement with the approved medical prescriptions of specific drugs to specific cancers. We argue that this analysis of knowledge accumulated in Wikipedia provides useful complementary global information about interdependencies between cancers, drugs and world countries.

## Introduction

“Nearly every family in the world is touched by cancer, which is now responsible for almost one in six deaths globally” [1]. The number of new cancer cases in the world is steadily growing reaching 18.1 million projected for 2018 [2] with predicted new cases of 29.4 million for 2035 [3]. The detailed statistical analysis of new cases and mortality projected for 2018 is reported in [4]. Such statistical analysis is of primary importance for estimating the influence of cancer diseases on the world population. However, it requires significant efforts of research groups and medical teams all over the world such as consortia involved in the Global Burden of Diseases Study (GBD) [5] and the WHO GLOBOCAN reports [2].

Here we propose to probe the network of Wikipedia articles in order to infer specific interactions between cancer types and to measure world influence of cancers. Wikipedia can be seen as a global database of accumulated human knowledge with an immense variety of topics. Moreover the way Wikipedia articles are citing each other encodes scientific, social, historical, and many other aspects. In principle one should be able to extract from Wikipedia direct or indirect relations between cancers, drug cancers and countries. We focus our study on cancer which is one of the major cause of human mortality and which consequently have important social and political impacts all around the world. We aim to measure these impacts through the prism of Wikipedia.

Thus we develop a complementary approach to the existing statistical approaches [2, 4, 5], the Wikipedia network analysis based on the Google matrix and PageRank algorithm invented by Brin and Page in 1998 for World Wide Web search engine information retrieval [6, 7]. Applications of this approach to various directed networks are described at [8]. Here we use the network of English Wikipedia articles collected in May 2017 with *N* = 5 416 537 articles and connected by *N*_*l*_ = 122 232 032 directed links, i.e. quotations from one article to another.

At present Wikipedia represents a public, open, collectively created encyclopaedia with a huge amount of information exceeding those of Encyclopedia Britannica [9] in volume and accuracy of articles devoted to scientific topics [10]. As an example, articles on biomolecules are actively maintained by Wikipedians [11, 12]. The academic analysis of information collected in Wikipedia is growing, getting more tools and applications as reviewed in [13, 14]. The scientific analysis shows that the quality of Wikipedia articles is growing [15].

A new element of our analysis is the reduced Google matrix (REGOMAX) method developed recently [16, 17]. This method selects a modest size subset of *N*_*r*_ nodes of interest from a huge global directed network with *N* ≫ *N*_*r*_ nodes and generates the reduced Google matrix *G*_R_ taking into account all direct pathways and indirect pathways (i.e. those going through the global network) between the *N*_*r*_ nodes. This approach conserves the PageRank probabilities of nodes from the global Google matrix *G* (up to a normalization factor). This method uses the ideas coming from the scattering theory of complex nuclei, mesoscopic physics and quantum chaos.

The efficiency of this approach has been tested with Wikipedia networks of politicians [17], painters [18], world universities [19], with biological networks from SIGNOR data base [20], with world trade networks [21, 22], and with financial networks [23]. The method is general as it can be applied to any subset of nodes embedded in a huge directed network. The main outcome is a synthetic effective view of the subnetwork encoded by weighted links in the corresponding reduced Google matrix. The strength of the specific application of REGOMAX method to Wikipedia networks is the encyclopedic nature of Wikipedia. Myriads of subjects are treated in Wikipedia which allow through the network of articles to connect, at least indirectly, many very different topics such as, e.g., for the present study, countries and cancer types. Moreover in the framework of the REGOMAX method every articles in Wikipedia, even articles having apparently nothing to do with the subjects of interest possibly contribute to the effective link obtained between two chosen nodes (i.e., articles). Although the quality of Wikipedia articles is constantly growing [10–12, 15], the information extraction may be sensitive to noise coming from inadequate or not so relevant links introduced in certain Wikipedia articles. These noisy links, which depends on when the Wikipedia network has been extracted, usually are cleaned out by the Wikipedians collaborative effort. The lifetime before removal of these noisy links depend also on the subject. As there is no simple way to quantify this source of noise (the degree of relevance of a link between two articles), we assume that in average it causes no harm to the present study. The results are presented in the devoted section keeping in mind this limitation.

In this work the reduced network is composed of *N*_*cr*_ = 37 types of cancers listed at Wikipedia [24] and *N*_*d*_ = 203 drugs for cancer extracted from data base [25]. All these *N*_*cr*_ + *N*_*d*_ = 240 items had an active Wikipedia article in May 2017. All these cancers and drugs are listed in alphabetic order in Tables 1 and 2. In addition we add to the selected set of articles *N*_*cn*_ = 195 world countries that allows us to analyze the global influence of cancer types (the ranking and REGOMAX analysis of countries are reported in [26, 27]). The PageRank list of the 195 selected countries is available at [28]. Thus in total the reduced Google matrix selected number of nodes is *N*_*r*_ = *N*_*cr*_ + *N*_*d*_ + *N*_*cn*_ = 435. The inclusion of these three groups (cancer types, cancer drugs, and countries) in the reduced set of *N*_*r*_ articles allows to investigate the interactions and influence of nodes inside group and between groups.

**Table 1:** List of articles devoted to cancer types in May 2017 English Wikipedia. This list of *N*_*cr*_ = 37 cancers taken from [24] is ordered by alphabetical order.

	Cancer type		Cancer type
1	Adrenal tumor	21	Mesothelioma
2	Anal cancer	22	Multiple myeloma
3	Appendix cancer	23	Neuroendocrine tumor
4	Bladder cancer	24	Non-Hodgkin lymphoma
5	Bone tumor	25	Oral cancer
6	Brain tumor	26	Ovarian cancer
7	Breast cancer	27	Pancreatic cancer
8	Cervical cancer	28	Prostate cancer
9	Cholangiocarcinoma	29	Skin cancer
10	Colorectal cancer	30	Soft-tissue sarcoma
11	Esophageal cancer	31	Spinal tumor
12	Gallbladder cancer	32	Stomach cancer
13	Gestational trophoblastic disease	33	Testicular cancer
14	Head and neck cancer	34	Thyroid cancer
15	Hodgkin’s lymphoma	35	Uterine cancer
16	Kidney cancer	36	Vaginal cancer
17	Leukemia	37	Vulvar cancer
18	Liver cancer		
19	Lung cancer		
20	Melanoma		

**Table 2:** List of articles devoted to cancer drugs in May 2017 English Wikipedia. This list of *N*_*d*_ = 203 cancer drugs taken from [25] is ordered by alphabetical order.

	Cancer drug		Cancer drug		Cancer drug		Cancer drug
1	Abemaciclib	52	Dactinomycin	103	Ixazomib	154	Prednisone
2	Abiraterone acetate	53	Daratumumab	104	Lanreotide	155	Procarbazine
3	Acalabrutinib	54	Dasatinib	105	Lapatinib	156	Propranolol
4	Afatinib	55	Daunorubicin	106	Lenalidomide	157	Protein-bound paclitaxel
5	Aflibercept	56	Decitabine	107	Lenvatinib	158	Radium-223
6	Alectinib	57	Defibrotide	108	Letrozole	159	Raloxifene
7	Alemtuzumab	58	Degarelix	109	Leuprorelin	160	Ramucirumab
8	Amifostine	59	Denileukin diftitox	110	Lomustine	161	Rasburicase
9	Aminolevulinic acid	60	Denosumab	111	Megestrol acetate	162	Regorafenib
10	Anastrozole	61	Dexamethasone	112	Melphalan	163	Ribociclib
11	Apalutamide	62	Dexrazoxane	113	Mercaptopurine	164	Rituximab
12	Aprepitant	63	Dinutuximab	114	Mesna	165	Rolapitant
13	Arsenic trioxide	64	Docetaxel	115	Methotrexate	166	Romidepsin
14	Asparaginase	65	Doxorubicin	116	Methylnaltrexone	167	Romiplostim
15	Atezolizumab	66	Durvalumab	117	Midostaurin	168	Rucaparib
16	Avelumab	67	Elotuzumab	118	Mitomycin C	169	Ruxolitinib
17	Axicabtagene ciloleucel	68	Eltrombopag	119	Mitoxantrone	170	Siltuximab
18	Axitinib	69	Enzalutamide	120	Necitumumab	171	Sipuleucel-T
19	Azacitidine	70	Epirubicin	121	Nelarabine	172	Sonidegib
20	Belinostat	71	Eribulin	122	Neratinib	173	Sorafenib
21	Bendamustine	72	Erlotinib	123	Netupitant/palonosetron	174	Sunitinib
22	Bevacizumab	73	Etoposide	124	Nilotinib	175	Talc
23	Bexarotene	74	Everolimus	125	Nilutamide	176	Talimogene laherparepvec
24	Bicalutamide	75	Exemestane	126	Niraparib	177	Tamoxifen
25	Bleomycin	76	Filgrastim	127	Nivolumab	178	Temozolomide
26	Blinatumomab	77	Fludarabine	128	Obinutuzumab	179	Temsirolimus
27	Bortezomib	78	Fluorouracil	129	Ofatumumab	180	Thalidomide
28	Bosutinib	79	Flutamide	130	Olaparib	181	ThioTEPA
29	Brentuximab vedotin	80	Folinic acid	131	Olaratumab	182	Tioguanine
30	Brigatinib	81	Fulvestrant	132	Omacetaxine mepesuccinate	183	Tipiracil
31	Busulfan	82	Gefitinib	133	Ondansetron	184	Tisagenlecleucel
32	Cabazitaxel	83	Gemcitabine	134	Osimertinib	185	Tocilizumab
33	Cabozantinib	84	Gemtuzumab ozogamicin	135	Oxaliplatin	186	Topotecan
34	Capecitabine	85	Glucarpidase	136	Paclitaxel	187	Toremifene
35	Carboplatin	86	Goserelin	137	Palbociclib	188	Trabectedin
36	Carfilzomib	87	HPV vaccines	138	Palifermin	189	Trametinib
37	Carmustine	88	Hyaluronidase	139	Palonosetron	190	Trastuzumab
38	Ceritinib	89	Hydroxycarbamide	140	Pamidronic acid	191	Trastuzumab emtansine
39	Cetuximab	90	Ibritumomab tiuxetan	141	Panitumumab	192	Trifluridine
40	Chlorambucil	91	Ibrutinib	142	Panobinostat	193	Uridine triacetate
41	Chlormethine	92	Idarubicin	143	Pazopanib	194	Valrubicin
42	Cisplatin	93	Idelalisib	144	Pegaspargase	195	Vandetanib
43	Cladribine	94	Ifosfamide	145	Pegfilgrastim	196	Vemurafenib
44	Clofarabine	95	Imatinib	146	Peginterferon	197	Venetoclax
45	Cobimetinib	96	Imiquimod	147	Pembrolizumab	198	Vinblastine
46	Copanlisib	97	Inotuzumab ozogamicin	148	Pemetrexed	199	Vincristine
47	Crizotinib	98	Interferon alfa-2b	149	Pertuzumab	200	Vinorelbine
48	Cyclophosphamide	99	Interleukin 2	150	Plerixafor	201	Vismodegib
49	Cytarabine	100	Ipilimumab	151	Pomalidomide	202	Vorinostat
50	Dabrafenib	101	Irinotecan	152	Ponatinib	203	Zoledronic acid
51	Dacarbazine	102	Ixabepilone	153	Pralatrexate		

The paper is composed as follows: the section “Description of data sets and methods” will present the May 2017 English Wikipedia network, introduce the Google matrix, the PageRank and CheiRank algorithms, and explain the construction of reduced Google matrices. In this section the node influence is defined through the PageRank ranking and the PageRank sensitivity. In the section “Results” we present the influence of cancer devoted pages in Wikipedia and extract a cancer ranking which is compared to cancer rankings extracted from GBD study [5] and GLOBOCAN [2] databases. We also use the reduced Google matrix to construct a reduced network of cancers and we determine the interaction of cancers with countries and cancer drugs. We corroborate the results obtained from the network structure of Wikipedia articles with various disease burden and/or epidemiological studies. To our knowledge, it is the first time that such correspondence is established. Finally we compare cancer prescriptions obtained from May 2017 English Wikipedia network analysis with approved medications reported in National Cancer Institute [25] and DrugBank [29]. The last section presents the conclusion of this research.

## Description of data sets and methods

### Network of English Wikipedia articles of 2017

**Fig 1:**
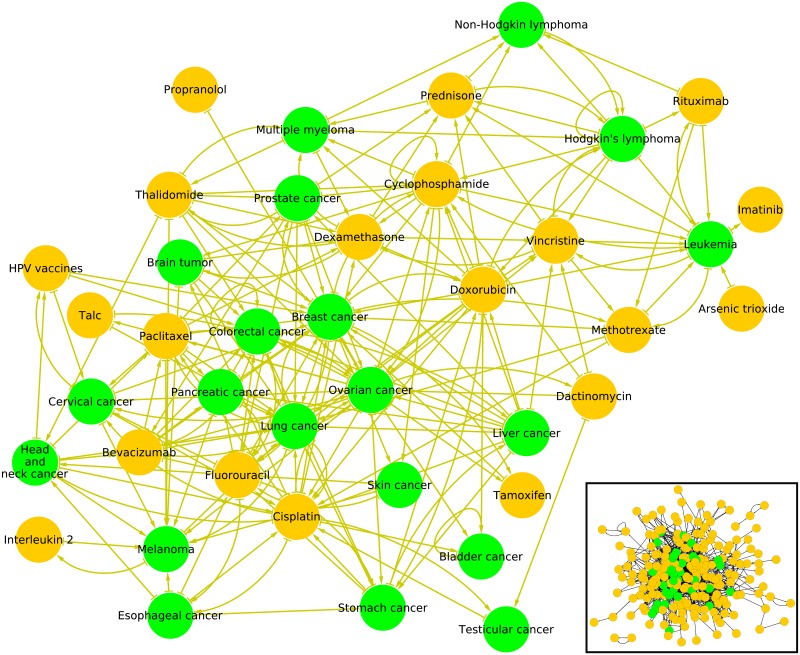
Subnetworks of cancers and cancer drugs in May 2017 English Wikipedia. Bottom right inset: subnetwork of *N*_*r*_ = 240 articles comprising *N*_*cr*_ = 37 articles devoted to cancers (green nodes) and *N*_*d*_ = 203 articles devoted to cancer drugs (golden nodes). Main figure: subnetwork of top 20 cancers and top 20 cancer drugs extracted from the ranking of 2017 English Wikipedia using PageRank algorithm (see Table 3). The bulk of the other Wikipedia articles is not shown. Arrows symbolize hyperlinks between cancer and cancer drug articles in the global Wikipedia.

We analyze the English language edition of Wikipedia collected in May 2017 (ENWIKI2017) [30] containing *N* = 5 416 537 articles (nodes) connected by *N*_*l*_ = 122 232 932 directed hyperlinks between articles (without self-citations). From this data set we extract the *N*_*cr*_ = 37 types of cancers listed at [24]. From [25] we also collect names of drugs related to cancer diseases obtaining the list of *N*_*d*_ = 203 drugs present at Wikipedia. The lists of 37 cancer types and 203 drugs are given in Table 1 and 2. This reduced set of *N*_*r*_ = 240 nodes is illustrated in the inset of Fig 1. For global influence investigations, it is complemented by *N*_*cn*_ = 195 world countries listed in [28]. Thus in total we have the reduced network of *N*_*r*_ = *N*_*cr*_ + *N*_*d*_ + *N*_*cn*_ = 435 ≪ *N* nodes embedded in the global network with more than 5 millions nodes. All data sets are available at [28]. The present study complies with Wikimedia terms of use.

### Google matrix construction rules

The construction rules of Google matrix *G* are described in detail in [6–8]. Thus the Google matrix *G* is built from the adjacency matrix *A*_*ij*_ with elements 1 if article (node) *j* points to article (node) *i* and zero otherwise. The Google matrix elements have the standard form *G*_*ij*_ = *αS*_*ij*_ + (1 − *α*)/*N* [6–8], where *S* is the matrix of Markov transitions with elements *S*_*ij*_ = *A*_*ij*_/*k*_*out*_(*j*). Here $k_{out}\left(j\right)=\sum_{i=1}^{N}A_{ij}\neq 0$ is the out-degree of node *j* (number of outgoing links) and *S*_*ij*_ = 1/*N* if *j* has no outgoing links (dangling node). The parameter 0 < *α* < 1 is the damping factor. For a random surfer, jumping from one node to another, it gives the probability (1 − *α*) to jump to any node. Below we use the standard value *α* = 0.85 [7] noting that for the range 0.5 ≤ *α* ≤ 0.95 the results are not sensitive to *α* [7, 8].

The right PageRank eigenvector of *G* is the solution of the equation *GP* = λ*P* with the unit eigenvalue λ = 1. The PageRank components *P*(*j*) give positive probabilities to find a random surfer on a node *j* after an infinite journey (∑_*j*_ *P*(*j*) = 1). The numerical computation of *P*(*j*) is done efficiently with the PageRank algorithm described in [6, 7].

The node influence is measured from the PageRank algorithm. We sort network nodes by decreasing PageRank probabilities. We assign *K* = 1 index to the node with maximal probability, i.e., the most central node according to PageRank algorithm, *K* = 2 index to the node with the second biggest probability, …A recursive definition of the PageRank algorithm can be given: a node is all the more influential as it is pointed by influential nodes.

It is also useful to consider the network with inverted direction of links. After links inversion $A_{ij}^{*}=A_{ji}$, the Google matrix G* is constructed within the same procedure with G*P* = P*. The matrix G* has its own PageRank vector P* called CheiRank [31] (see also [8, 32]). Its probability values can be again ordered in a decreasing order with CheiRank index K* with highest P*(*j*) at K* = 1 and smallest at K* = *N*. The CheiRank algorithm measures the node diffusivity. A recursive definition of the CheiRank algorithm can also be given: a node is all the more diffusive as it is pointed by diffusive nodes.

On average, the high values of *P*(*j*) (P*(*j*)) correspond to nodes *j* with many ingoing (outgoing) links [8].

**Fig 2:**
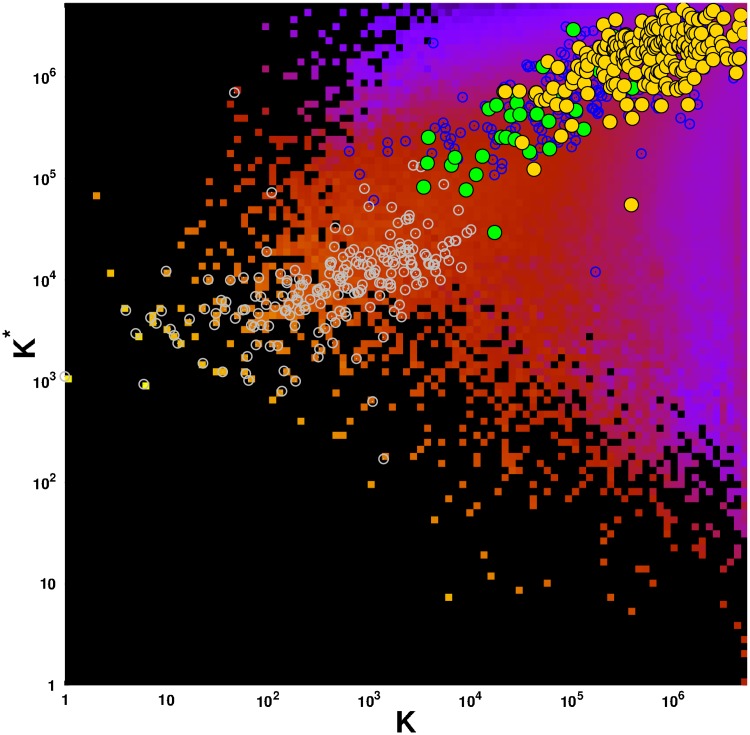
Density of May 2017 English Wikipedia articles in the CheiRank K*—PageRank *K* plane. Data are averaged over a 100 × 100 grid spanning the (log_10_ *K*, log_10_ K*) ∈[0, log_10_ *N*] × [0, log_10_ *N*] domain. Density of articles ranges from very low density (purple tiles) to very high density (bright yellow tiles). The absence of article is represented by black tiles. The superimposed green (gold) circles give the positions of May 2017 English Wikipedia articles devoted to cancers (cancer drugs) listed in Table 1 (Table 2). For comparison, the gray (blue) open circles give the positions of pages devoted to sovereign countries (infectious diseases) in May 2017 English Wikipedia.

The PageRank order list of 37 cancers and 203 drugs is given in Table 3. In the global ENWIKI2017 network, countries are located on top PageRank positions (1. *USA*, 4. *France*, 5. *Germany*) so that cancers and drugs are located well below them since the first cancer type, i.e. *Lung cancer*, appears at 3 478th position, and the first cancer drug, i.e. *Talc*, appears at 22 177th position (see Fig 2). As expected cancer types have a more central position than cancer drugs. The network of 40 nodes and their direct links is shown in Fig 1 for the top 20 PageRank nodes of cancers and drugs (ordered separately for cancers and drugs). We see that already only for 40 nodes the network structure is rather complex. Here and below the networks are drawn with Cytoscape [33].

**Table 3:** Ranking of articles devoted to cancer types and to cancer drugs in May 2017 English Wikipedia using PageRank algorithm. Cancer types are highlighted in boldface.

*K*_*r*_	*K*_*cr*_	*K*_*d*_	Cancer/drug	*K*_*r*_	*K*_*cr*_	*K*_*d*_	Cancer/drug	*K*_*r*_	*K*_*cr*_	*K*_*d*_	Cancer/drug	*K*_*r*_	*K*_*cr*_	*K*_*d*_	Cancer/drug	*K*_*r*_	*K*_*d*_	Drug
1	**1**		**Lung**	49		22	Trastuzumab	97		65	Mitoxantrone	145		110	Eribulin	193	156	Ixazomib
2	**2**		**Breast**	50		23	Vinblastine	98	**33**		**Gallbladder**	146		111	Panitumumab	194	157	Lenvatinib
3	**3**		**Leukemia**	51	**28**		**NETs** ^c^	99		66	Vemurafenib	147		112	Ofatumumab	195	158	Trifluridine
4	**4**		**Prostate**	52		24	Bleomycin	100		67	Topotecan	148	**36**		**Adrenal**	196	159	Ponatinib
5	**5**		**Colorectal**	53		25	Carboplatin	101		68	Fludarabine	149		113	Sipuleucel-T	197	160	Alectinib
6	**6**		**Brain**	54		26	Mercaptopurine	102		69	Pembrolizumab	150		114	Pamidronic	198	161	Nilutamide
7	**7**		**Pancreatic**	55		27	Docetaxel	103		70	Tioguanine	151		115	Cabozantinib	199	162	Daratumumab
8	**8**		**Melanoma**	56		28	Daunorubicin	104		71	Dacarbazine	152		116	Brentuximab	200	163	Valrubicin
9	**9**		**Stomach**	57		29	Hyaluronidase	105		72	Azacitidine	153		117	Gemtuzumab	201	164	Sonidegib
10	**10**		**Ovarian**	58		30	Etoposide	106	**34**		**Vaginal**	154		118	Enzalutamide	202	165	Osimertinib
11	**11**		**Cervical**	59		31	Bortezomib	107		73	Carmustine	155		119	Pegfilgrastim	203	166	Pertuzumab
12	**12**		**Hodgkin’s**	60		32	Irinotecan	108		74	Decitabine	156		120	Romidepsin	204	167	Defibrotide
13	**13**		**Skin**	61	**29**		**Soft-tissue**	109		75	Bicalutamide	157		121	Rasburicase	205	168	Bexarotene
14		1	Talc	62		33	Oxaliplatin	110		76	Flutamide	158		122	Bendamustine	206	169	Palifermin
15	**14**		**M. myeloma**	63		34	Melphalan	111	**35**		**Vulvar**	159		123	Interferon	207	170	Idelalisib
16	**15**		**Esophageal**	64		35	Leuprorelin	112		77	Procarbazine	160		124	Obinutuzumab	208	171	Toremifene
17	**16**		**Liver**	65		36	Raloxifene	113		78	Cladribine	161		125	Denileukin	209	172	Apalutamide
18	**17**		**Non-Hodgkin**	66		37	Hydroxycarb.^d^	114		79	Tocilizumab	162		126	Ruxolitinib	210	173	Regorafenib
19	**18**		**Bladder**	67		38	Aminolevulinic	115		80	Busulfan	163		127	Talimogene	211	174	Venetoclax
20		2	Methotrexate	68		39	Cytarabine	116		81	Denosumab	164		128	Belinostat	212	175	Dexrazoxane
21	**19**		**Head & Neck**	69		40	Cetuximab	117		82	Pemetrexed	165		129	Eltrombopag	213	176	Avelumab
22		3	Thalidomide	70		41	Folinic acid	118		83	Lomustine	166		130	Cabazitaxel	214	177	Dinutuximab
23	**20**		**Testicular**	71		42	Mitomycin C	119		84	Vinorelbine	167		131	Lanreotide	215	178	Ramucirumab
24		4	Paclitaxel	72	**30**		**Anal**	120		85	Nivolumab	168		132	Palbociclib	216	179	Blinatumomab
25		5	Prednisone	73		43	Gemcitabine	121		86	Dabrafenib	169		133	Pomalidomide	217	180	Rolapitant
26		6	Cisplatin	74		44	Sorafenib	122		87	Letrozole	170		134	Trastuzumab	218	181	Niraparib
27		7	Dexamethasone	75		45	Imiquimod	123		88	Fulvestrant	171		135	Vismodegib	219	182	Pralatrexate
28	**21**		**Thyroid**	76	**31**		**Spinal**	124		89	Radium-223	172	**37**		**Appendix**	220	183	Acalabrutinib
29		8	Doxorubicin	77		46	Sunitinib	125		90	Olaparib	173		136	Omacetaxine	221	184	Brigatinib
30	**22**		**Bone**	78		47	Ifosfamide	126		91	Pazopanib	174		137	Plerixafor	222	185	Necitumumab
31		9	Propranolol	79		48	Erlotinib	127		92	Dasatinib	175		138	Lapatinib	223	186	Midostaurin
32		10	Interleukin 2	80		49	Asparaginase	128		93	Idarubicin	176		139	Clofarabine	224	187	Rucaparib
33	**23**		**Kidney**	81		50	Gefitinib	129		94	Temsirolimus	177		140	Vandetanib	225	188	Inotuzumab
34	**24**		**Mesothelioma**	82	**32**		**GTD** ^e^	130		95	Exemestane	178		141	Axitinib	226	189	Pegaspargase
35		11	Cyclophospha.^a^	83		51	Anastrozole	131		96	Crizotinib	179		142	Ibrutinib	227	190	Durvalumab
36		12	Fluorouracil	84		52	Epirubicin	132		97	Zoledronic	180		143	Methylnal^g^	228	191	Siltuximab
37	**25**		**Oral**	85		53	Lenalidomide	133		98	Panobinostat	181		144	Carfilzomib	229	192	Ribociclib
38		13	Tamoxifen	86		54	Capecitabine	134		99	Mesna	182		145	Protein-bound	230	193	Degarelix
39		14	Vincristine	87		55	Vorinostat	135		100	Ibritumomab	183		146	Bosutinib	231	194	Neratinib
40		15	Rituximab	88		56	Chlormethine	136		101	Trametinib	184		147	Ceritinib	232	195	Abemaciclib
41		16	Bevacizumab	89		57	Everolimus	137		102	Nilotinib	185		148	Abiraterone	233	196	Olaratumab
42		17	HPV vaccines	90		58	Alemtuzumab	138		103	Ixabepilone	186		149	Trabectedin	234	197	Copanlisib
43		18	Imatinib	91		59	Chlorambuci.^f^	139		104	Megestrol	187		150	Elotuzumab	235	198	Netupitant
44		19	Arsenic trioxide	92		60	Filgrastim	140		105	Romiplostim	188		151	Nelarabine	236	199	Tipiracil
45	**26**		**Uterine**	93		61	Goserelin	141		106	Afatinib	189		152	Palonosetron	237	200	Uridine
46		20	Dactinomycin	94		62	Ipilimumab	142		107	ThioTEPA	190		153	Cobimetinib	238	201	Axicabtagene
47	**27**		**Cholangio**.^b^	95		63	Temozolomide	143		108	Aprepitant	191		154	Amifostine	239	202	Glucarpidase
48		21	Ondansetron	96		64	Peginterferon	144		109	Aflibercept	192		155	Atezolizumab	240	203	Tisagenlecleucel

Notes: here words “cancer”, “tumor”, “lymphoma”, “sarcoma” have been removed from cancer type denominations;

^a^Cyclophosphamide;

^b^Cholangiocarcinoma;

^c^Neuroendocrine tumors;

^d^Hydroxycarbamide;

^e^Gestational trophoblastic disease;

^f^Chlorambucil;

^g^Methylnaltrexone.

### Reduced Google matrix algorithm

The details of REGOMAX method are described in [16, 17, 20]. It captures in the reduced Google matrix of size *N*_*r*_ × *N*_*r*_ the full contribution of direct and indirect pathways existing in the full Google matrix between *N*_*r*_ nodes of interest. The reduced Google matrix *G*_R_ is such as *G*_R_*P*_*r*_ = *P*_*r*_ where *P*_*r*_ is its associated PageRank probability vector. The PageRank probabilities *P*_*r*_(*j*) of the selected *N*_*r*_ nodes are the same as for the global network with *N* nodes, up to a constant multiplicative factor taking into account that the sum of PageRank probabilities over *N*_*r*_ nodes is unity. The computation of *G*_R_ provides a decomposition into matrices that clearly distinguish direct from indirect interactions: *G*_R_ = *G*_*rr*_ + *G*_pr_ + *G*_qr_ [17]. Here *G*_*rr*_ is the *N*_*r*_ × *N*_*r*_ submatrix of the *N* × *N* global Google matrix *G* encoding the direct links between the selected *N*_*r*_ nodes. The G_pr_ matrix is rather close to the matrix in which each column is given by the PageRank vector *P*_*r*_, ensuring that PageRank probabilities of *G*_R_ are the same as for *G* (up to a constant multiplier). Thus G_pr_ does not provide much more information about direct and indirect links between selected nodes than the usual Google matrix analysis described in the previous section. The component playing an interesting role is *G*_qr_, which takes into account all indirect links between selected nodes appearing due to multiple paths via the global network of *N* nodes (see [16, 17]). The matrix *G*_qr_ = *G*_qrd_ + *G*_qrnd_ has diagonal (*G*_qrnd_) and non-diagonal (*G*_qrnd_) parts. Thus *G*_qrnd_ describes indirect interactions between nodes. The explicit formulas as well as the mathematical and numerical computation methods of all three components of *G*_R_ are given in [16, 17, 20].

With the reduced Google matrix *G*_R_ and its components we can analyze the PageRank sensitivity in respect to specific links between *N*_*r*_ nodes. To measure the sensitivity of a country *cn* to a cancer *cr* we change the matrix element (*G*_*R*_)_*cn*,*cr*_ by a factor (1+*δ*) with *δ* ≪ 1 and renormalize to unity the sum of the column elements associated with cancer *cr*, and we compute the logarithmic derivative of PageRank probability *P*(*cn*) associated to country *cn*: *D*(*cr* → *cn*, *cn*) = *d* ln *P*(*cn*)/*dδ* (diagonal sensitivity). It is also possible to consider the nondiagonal (or indirect) sensitivity *D*(*cr* → *cn*, *cn*′) = *d* ln *P*(*cn*′)/*dδ* when the variation is done for the link from *cr* to *cn* and the derivative of PageRank probability is computed for another country *cn*′. Also instead of the link *cr* → *cn* we can consider the link from a cancer *cr* to a drug *d* computing then the nondiagonal sensitivity of country *cn*′. The PageRank sensitivity approach, already used in [26, 27], allows to measure the sensitivity of a node influence with respect to the change of a link intensity. Pragmatically it measures the increase or decrease of influence of a node *A* caused by an increase of the intensity of the link from a node *B* to a node *C*.

## Results

### Cancer distribution on PageRank-CheiRank plane

The PageRank order of 37 cancers and 203 cancer drugs is given in Table 3. In the top 3 positions we find *Lung, Breast, Leukemia* cancers. *Lung* and *Breast cancers* have indeed the two highest incidences [2] and *Leukemia* is the most frequent type of cancer in children and young adults [34]. In general in the PageRank order of 240 cancers and drugs, cancers occupy predominantly the top positions. The first three drugs are *Talc, Methotrexate, Thalidomide*, taking positions 14, 20, 22. The top position of *Talc* among cancer drugs may be explained by its industrial use and also by both potential carcinogenic and anticancer effects [35]. *Methotrexate* can be used in the most frequent types of cancer but also in autoimmune diseases and for medical abortions [36]. The third position of *Thalidomide* among cancer drugs may be explained by its high potential for the treatment of cancers but also for its well-known teratogenic effect; this teratogenic effect may by itself contribute to its prominence in Wikipedia. It is also used for treatment of other diseases than cancers (tuberculosis, graft-versus-host disease,…) [37]. The list of these 240 articles in CheiRank order is also given in [28].

The distribution of selected articles on the global PageRank-CheiRank plane of the whole Wikipedia network with *N* = 5 416 537 nodes are shown in Fig 2. The top PageRank positions are taking by the world countries as discussed in [8, 26] marked by gray open circles. Then there is a group of cancers (above *K* ∼ 3 × 10^3^ and K* ∼ 10^4^), marked by green points, followed by drugs (mostly above *K* ∼ 10^4^ and K* ∼ 10^5^), marked by gold points. There is a certain overlap between cancers and drugs on this plane but in global there is a clear separation between these two groups. As a comparison we also mark the positions of 230 infectious diseases by open blue circles. These 230 articles are studied in [27] in the frame of Wikipedia network analysis. The global PageRank list of 230 infectious diseases and 37 cancers is given in [28]. In this list *Lung cancer* is located at the 7th position. From Fig 2 we observe these two types of diseases occupy somewhat the same (*K*, K*) region (mostly above K* ∼ 10^5^ and above *K* ∼ 3 × 10^3^) suggesting that cancer types and infectious diseases have globally the same influence in May 2017 English Wikipedia with the exception of the first six infectious diseases, *Tuberculosis* (*K* = 639), *HIV/AIDS* (*K* = 810), *Malaria* (*K* = 1116), *Pneumonia* (*K* = 1531), *Smallpox* (*K* = 1532), *Cholera* (*K* = 2300) which are causing or have caused pandemics or notable epidemics. The first three cancer types, i.e. *Lung cancer*, *Breast cancer*, and *Leukemia*, appear at positions *K* = 3478, 3788, and 3871 just before *Influenza* at *K* = 4191.

**Fig 3:**
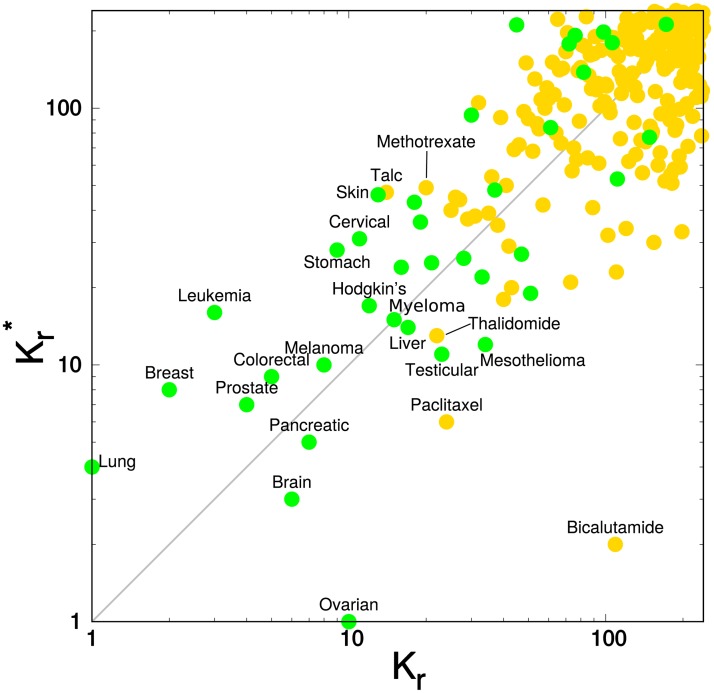
Distribution of the May 2017 English Wikipedia articles devoted to cancers and drug cancers in the local CheiRank $K_{r}^{*}$—PageRank *K*_*r*_ plane. The *N*_*cr*_ = 37 (*N*_*d*_ = 203) articles devoted to cancers (drug cancers) are represented by green (gold) plain circles.

The 240 cancer types and drugs placed on the plane of local PageRank indices *K*_*r*_ ∈ {1, …, 240} and CheiRank indices $K_{r}^{*}\in \left\{1,\ldots ,240\right\}$ is shown in Fig 3. We retrieve the fact that cancer types occupy the top positions in *K*_*r*_ and in $K_{r}^{*}$. Indeed the first 14 most influent articles of this subset (*K* ≤ 14), which appear to be devoted to cancer types, are also the most communicative with the exception of articles devoted to drugs *Paclitaxel* $\left(K_{r}=24,K_{r}^{*}=6\right)$ and *Bicalutamide* $\left(K_{r}=109,K_{r}^{*}=2\right)$. *Paclitaxel* [38] is a chemotherapy medication used to treat a wide range of cancer types e.g. *Ovarian cancer, Breast cancer, Lung cancer, Pancreatic cancer*, etc. Moreover *Paclitaxel* article cites *Ovarian cancer* article $\left(K_{r}=10,K_{r}^{*}=1\right)$ which is a very communicative article since the *Ovarian cancer* article CheiRank index, K* = 29 317, is about one order magnitude lower than the CheiRank indexes, K* ≳ 10^5^, of the other 239 considered articles (see Fig 2). The wide applications of *Paclitaxel* and the citation of *Ovarian cancer* article explain the very good ranking of this cancer drug in the CheiRank scale. On the other hand, the $K_{r}^{*}=2$ rank of the *Bicalutamide* article (see Fig 3), devoted to an antiandrogen medication mainly used to treat *Prostate cancer*, is due to a very long article with a high density of intra-wiki citations [39]. Like the *Paclitaxel* article, the *Bicalutamide* article cites also the *Ovarian cancer* since this medication has already been tried for this cancer type [39].

The three most influent cancer drugs in ENWIKI2017 are *Talc*, *K*_*r*_ = 14, which is used to prevent blood effusions, e.g., in *Lung cancer* or *Ovarian cancer* [35], *Methotrexate*, *K*_*r*_ = 20, which is a chemotherapy agent used for the treatment *Breast cancer, Leukemia, Lung cancer, Lymphoma*, etc [36], and *Thalidomide*, *K*_*r*_ = 22, which is a drug modulating the immune system used, e.g., for *Multiple myeloma* treatment [37]. Although *Talc* is widely used in chemical, pharmaceutical and food industries [35], its global PageRank position is nevertheless of the same order than the PageRank position of the second most influent cancer drug in Wikipedia, i.e., *Methotrexate*, which is a drug more specific to cancers [36].

### Comparison of Wikipedia network analysis with GBD study 2017 and GLOBOCAN 2018 for cancer significance

**Fig 4:**
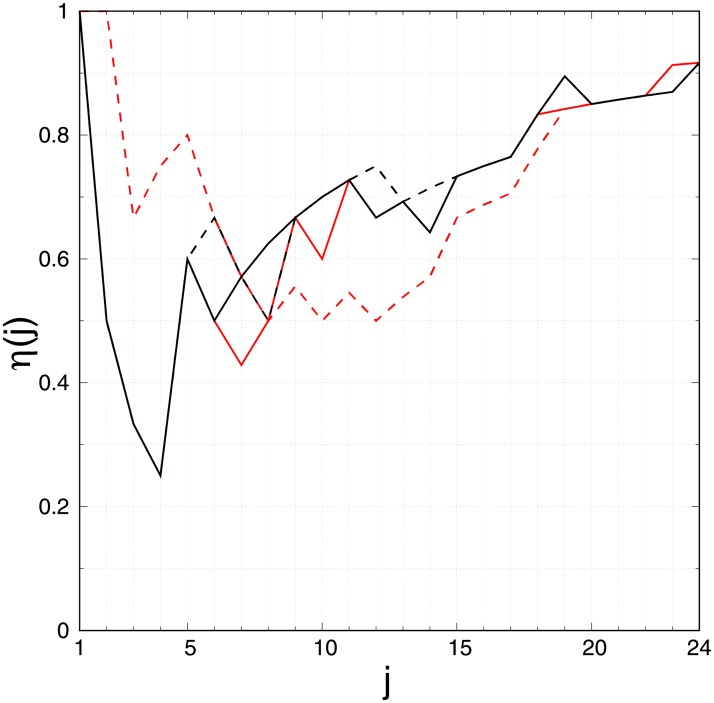
Comparison between cancer rankings extracted from May 2017 English Wikipedia PageRank, from the global burden of disease (GBD) study 2017 data, and from GLOBOCAN 2018 data. The overlap *η*(*j*) gives the number of cancer types in common in the top *j* of the ranking of cancers obtained from the May 2017 English Wikipedia PageRank (see bold terms in Table 3) and in the top *j* of the ranking of cancers by estimated number of worldwide deaths from GBD 2017 data [40] (black line, see Table 4), by estimation of disability-adjusted life years from GBD 2017 data [41] (black dashed line, Table 4), by estimated number of worldwide deaths from GLOBOCAN 2018 data [4] (red line, Table 5), and by estimated number of new cases from GLOBOCAN 2018 data [4] (red dashed line, Table 5). Only the black plain line is visible, where black plain line, red plain line and black dashed line overlap, e.g., from *j* = 1 to *j* = 5.

We perform the comparison of cancer significance given by the GBD study 2017 [5], the GLOBOCAN 2018 [2], and the Wikipedia network analysis. We extract the rankings of cancer types by the number of deaths in 2017 estimated by the 2017 GBD study [40] (see Table 4) and by the number of disability-adjusted life years (DALYs) estimated by the 2017 GBD study [41] (see Table 4). Also, we extract the rankings of cancer types by the number of deaths and by the number of new cases in 2018 estimated by the GLOBOCAN 2018 [4] (see Table 5). In Fig 4, we show the overlap of these 4 rankings with the extracted ranking of cancer types obtained from the ENWIKI2017 PageRanking (see bold items in Table 3). We observe that the ranking obtained from the Wikipedia network analysis provides a reliable cancer types ranking since its top 10 (top 20) shares about 70% (80%) similarity with GBD study data and GLOBOCAN data. The Wikipedia top 5 reaches even 80% similarity with top 5 cancer types extracted from the estimated number of new cases in 2018.

**Table 4:** List of cancer types ordered by the estimated number of deaths during the year 2017 (A) and by the estimated disability-adjusted life years (DALYs) for 2017 (B). Data extracted from GBD Study [40, 41].

A			B		
Rank	Cancer	Deaths in 2017 (× 10^3^)	Rank	Cancer	DALYs in 2017 (× 10^3^)
1	Lung cancer	1883.1	1	Lung cancer	40900
2	Colorectal cancer	896.0	2	Liver cancer	20800
3	Stomach cancer	865.0	3	Stomach cancer	19100
4	Liver cancer	819.4	4	Colorectal cancer	19000
5	Breast cancer	611.6	5	Breast cancer	17700
6	Pancreatic cancer	441.1	6	Leukemia	12000
7	Esophageal cancer	436.0	7	Head and neck cancer	10600
8	Prostate cancer	415.9	8	Esophageal cancer	9780
9	Head and neck cancer	380.6	9	Pancreatic cancer	9080
10	Leukemia	347.6	10	Brain tumor	8740
11	Cervical cancer	259.7	11	Cervical cancer	8060
12	Non-Hodgkin lymphoma	248.6	12	Prostate cancer	7060
13	Brain tumor	247.1	13	Non-Hodgkin lymphoma	7020
14	Bladder cancer	196.5	14	Ovarian cancer	4670
15	Ovarian cancer	176.0	15	Bladder cancer	3600
16	Gallbladder cancer	174.0	16	Gallbladder cancer	3480
17	Kidney cancer	138.5	17	Kidney cancer	3280
18	Skin cancer	126.8	18	Skin cancer	2980
19	Multiple myeloma	107.1	19	Multiple myeloma	2330
20	Uterine cancer	85.2	20	Uterine cancer	2140
21	Thyroid cancer	41.2	21	Hodgkin’s lymphoma	1380
22	Hodgkin’s lymphoma	32.6	22	Thyroid cancer	1130
23	Mesothelioma	29.9	23	Mesothelioma	671
24	Testicular cancer	7.7	24	Testicular cancer	375

**Table 5:** List of cancer types ordered by the estimated number of deaths during the year 2018 (A) and by the estimated number of new cases in 2018 (B). Data extracted from GLOBOCAN 2018 [4].

A			B		
Rank	Cancer	Deaths in 2018 (× 10^3^)	Rank	Cancer	New cases in 2018 (× 10^3^)
1	Lung cancer	1761.0	1	Lung cancer	2093.9
2	Colorectal cancer	861.7	2	Breast cancer	2088.8
3	Stomach cancer	782.7	3	Colorectal cancer	1801.0
4	Liver cancer	781.6	4	Prostate cancer	1276.1
5	Breast cancer	626.7	5	Skin cancer	1042.1
6	Esophageal cancer	508.6	6	Stomach cancer	1033.7
7	Head and neck cancer	453.3	7	Head and neck cancer	887.7
8	Pancreatic cancer	432.2	8	Liver cancer	841.1
9	Prostate cancer	359.0	9	Esophageal cancer	572.0
10	Cervical cancer	311.4	10	Cervical cancer	569.8
11	Leukemia	309.0	11	Thyroid cancer	567.2
12	Non-Hodgkin lymphoma	248.7	12	Bladder cancer	549.4
13	Brain tumor	241.0	13	Non-Hodgkin lymphoma	509.6
14	Bladder cancer	199.9	14	Pancreatic cancer	458.9
15	Ovarian cancer	184.8	15	Leukemia	437.0
16	Kidney cancer	175.1	16	Kidney cancer	403.3
17	Gallbladder cancer	165.1	17	Uterine cancer	382.1
18	Multiple myeloma	106.1	18	Brain tumor	296.9
19	Uterine cancer	89.9	19	Ovarian cancer	295.4
20	Skin cancer	65.2	20	Melanoma	287.7
21	Melanoma	60.7	21	Gallbladder cancer	219.4
22	Thyroid cancer	41.1	22	Multiple myeloma	160.0
23	Hodgkin lymphoma	26.2	23	Hodgkin lymphoma	80.0
24	Mesothelioma	25.6	24	Testicular cancer	71.1

### Reduced Google matrix of cancers and drugs

**Fig 5:**
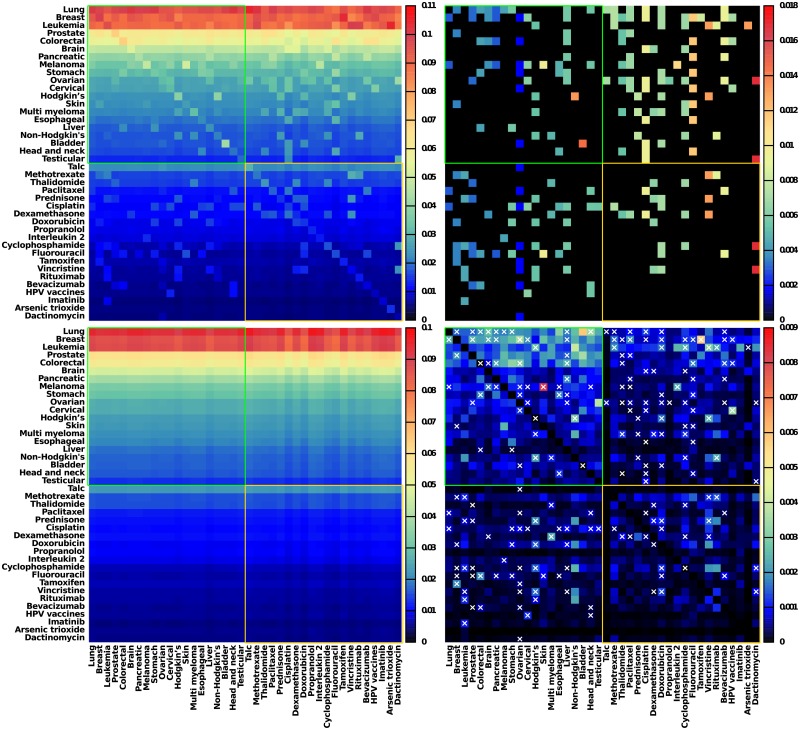
Reduced Google matrix *G*_R_ associated to the intertwined subnetworks of top 20 cancer articles and of top 20 drug articles. The reduced Google matrix *G*_R_ (top left) and its 3 components *G*_*rr*_ (top right), G_pr_ (bottom left), and *G*_qrnd_ (bottom right) are shown. The weights of the components are *W*_R_ = 1, *W*_pr_ = 0.872, *W*_*rr*_ = 0.086, and *W*_qr_ = 0.042 (*W*_qrnd_ = 0.038). For each component, thin green and gold lines delimit cancers and drugs sectors, i.e. upper left sub-matrix characterizes *from cancers to cancers* interactions, lower right sub-matrix *from drugs to drugs* interactions, upper right sub-matrix *from drugs to cancers* interactions, and lower left sub-matrix *from cancers to drugs* interactions. On the *G*_qrnd_ component (bottom right) superimposed crosses indicate links already present in the adjacency matrix (otherwise stated links corresponding to non zero entries in *G*_*rr*_, see top right).

Let us consider now the subset of *N*_*r*_ = 40 nodes composed of the first 20 cancers and the first 20 cancer drugs of the ENWIKI2017 PageRanking (Table 3). For this sub-network of interest illustrated in Fig 1, we perform the calculation of the reduced Google matrix *G*_R_ and its components *G*_*rr*_, G_pr_ and, G_qr_. From Fig 5, as expected, we observe that the *G*_R_ matrix (top left panel) is dominated by the G_pr_ component (bottom left panel) since *W*_pr_ = 0.872 *W*_R_. The G_pr_ component is of minor interest as it expresses again the relative PageRanking between the *N*_*r*_ = 40 cancers and drugs already obtained and discussed in previous sections. The *G*_*rr*_ (top right panel) gives the direct links between the considered cancers and drugs. Indeed, the *G*_*rr*_ matrix is similar to the adjacency matrix *A* since there is a one-to-one correspondence between non zero entries of *G*_*rr*_ and of *A* (for *G*_*rr*_ by non zero entry we mean an entry greater than (1 − *α*)/*N* ≃ 2.8 × 10^−8^). Fig 1 illustrates the subnetwork of the direct links between the top 20 cancer types and the top 20 cancer drugs encoded in *G*_*rr*_ and *A*. Once the obvious G_pr_ component and the direct links *G*_*rr*_ component removed from the reduced Google matrix *G*_R_, the remaining part G_qr_ gives the hidden links between the set of *N*_*r*_ nodes of interest. In Fig 5 we represent *G*_qrnd_ (bottom right panel), the non diagonal part of *G*_qr_. We can consider that a link with a non zero entry in *G*_qrnd_ and a zero entry in *G*_*rr*_ (consequently also in *A*) is a hidden link. Below we use the non obvious components of *G*_*rr*_ + *G*_qrnd_ to draw the structure of reduced network.

### Reduced network of cancers

**Fig 6:**
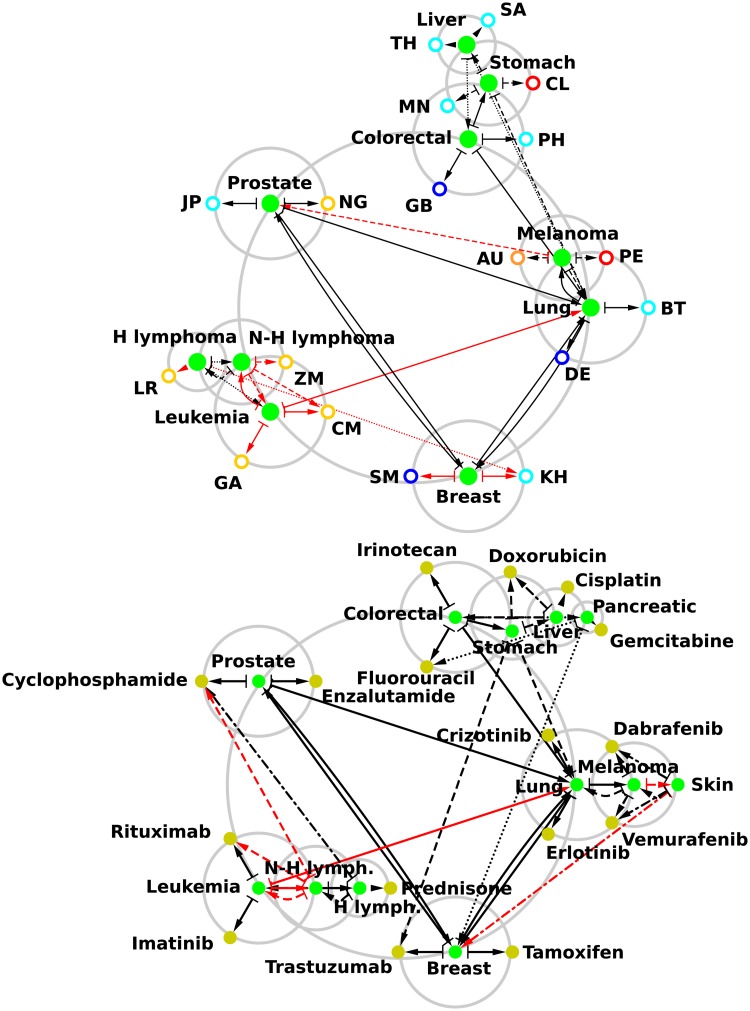
Reduced network of cancers. We consider the reduced Google matrix associated to the *N*_*cr*_ = 37 cancers and (top panel) the *N*_*cn*_ = 195 countries, (bottom panel) the *N*_*d*_ = 203 cancer drugs. We consider the top 5 cancers from the ranking of May 2017 English Wikipedia using the PageRank algorithm: 1. *Lung cancer*, 2. *Breast cancer*, 3. *Leukemia*, 4. *Prostate cancer*, 5. *Colorectal cancer* (see Table 3). These 5 cancers are symbolized by plain green nodes distributed around the central gray circle. We determine the two cancers to which each of these 5 cancers are preferentially linked according to (*G_rr_* + *G*_qrnd_). If not among the top 5 cancers, a newly determined cancer is placed on a gray circle centered on the cancer from which it is linked. Then for each one of the newly added cancers we determine the two best cancers to which they are each linked, and so on. This process is stopped once no new cancers can be added, i.e. at the 3rd iteration (top panel) and 4th iteration (bottom panel). Also, at each iteration the two countries (drugs) to which each cancer are preferentially linked are placed on the gray circle centered on the cancer; see top panel (bottom panel). No new links are determined from the newly added countries or drugs. On top panel, countries are represented by ring shaped nodes (red for American countries, yellow for African countries, cyan for Asian countries, blue for European countries, and orange for Oceanian countries). On bottom panel, drugs are represented by plain gold nodes. The arrows represent the directed links between cancers and from cancers to countries or drugs (1st iteration: plain line; 2nd iteration: dashed line; 3rd iteration: dotted line for top panel and dashed-dotted line for bottom panel; 4th iteration: dotted line for bottom panel). Black arrows correspond to links existing in the adjacency matrix, i.e., direct links, and red arrows are purely hidden links absent from the adjacency matrix but present in the *G*_qr_ component of the reduced Google matrix *G*_R_. These networks have been drawn with Cytoscape [33].

We construct the reduced Google matrix associated to the set of *N*_*r*_ = *N*_*cr*_ + *N*_*cn*_ = 232 Wikipedia articles constituted of *N*_*cr*_ = 37 articles devoted to cancer types and of *N*_*cn*_ = 195 articles devoted to countries. We consider the top 5 cancer types appearing in the ranking of May 2017 English Wikipedia using the PageRank algorithm which, according to Table 3, are 1 *Lung cancer*, 2 *Breast cancer*, 3 *Leukemia*, 4 *Prostate cancer*, 5 *Colorectal cancer*. Let us ordinate cancer types by their relative ranking in Table 3, cancer type *cr*_*i*_ is consequently the *i*th most influent cancer type in May 2017 English Wikipedia. Using the reduced Google matrix, the component ${\left(G_{rr}+G_{\text{qrnd}}\right)}_{cr_{i},cr_{j}}$, where *i*,*j* ∈ {1,…,*N*_*cr*_}, gives the non obvious strength of the link pointing from the *j*th to the *i*th most influent cancer types. From each one the top 5 cancer types, {*cr*_*j*_}_*j*∈{1,…,5}_, we select the two cancer types $cr_{i_{1}}$ and $cr_{i_{2}}$, with *i*_1_,*i*_2_ ∈ {1,…,*j* − 1,*j* + 1,…,*N*_*cr*_}, to which cancer type *cr*_*j*_ is preferentially linked (“friends”), i.e. those giving the two strongest ${\left(G_{rr}+G_{\text{qrnd}}\right)}_{cr_{i},cr_{j}}$ components. Around the main circle in Fig 6 (top panel) we first place the top 5 most influent cancer types in May 2017 English Wikipedia. Then we connect each one of these cancer types to their two above defined cancer type friends. If these cancer types are not yet present in the network we add them in the vicinity of the cancer type pointing them. For each newly added cancer type we reiterate the same process until no new cancer type is added to the reduced network. The construction process of the reduced network of cancer ends at the 3rd iteration (see Fig 6, top panel) exhibiting only 10 of the *N*_*cr*_ = 37 cancer types, which in addition of the top 5 cancer types, are 8 *Melanoma*, 9 *Stomach cancer*, 12 *Hodgkin lymphoma*, 17 *Liver cancer* and 18 *Non-Hodgkin lymphoma*. Among these 10 cancer types, 7 are among the top 10 deadliest in 2017 according to GBD study (see Table 4). In the reduced network of cancers showed in Fig 6 (top panel) we observe that the most influent cancer, i.e., *Lung cancer* is pointed from all the other cancer types with the exception of *Hodgkin and Non-Hodgkin lymphomas*. Also, Fig 6 (top panel) exhibits clearly a cluster of cancers (*Colorectal, Stomach*, and *Liver cancers*) affecting the digestive system, a cluster of cancers (*Hodgkin and Non-Hodgkin lymphomas*, and *Leukemia*) affecting blood, a loop interaction between *Prostate* and *Breast cancers* which are both linked to steroid hormone pathways and may be both treated with hormone therapy [42, 43], loop interactions between *Lung* and *Breast cancers* and between *Lung cancer* and *Melanoma* affecting mainly the thoracic region.

It is worth to note that although *Leukemia* article in May 2017 English Wikipedia does not cite any of the other articles devoted to cancer types (as an illustration the first half of the *Leukemia* column in *G*_*rr*_ is filled with zero entries, see Fig 5 top right panel), we are able to infer hidden links (in red in Fig 6, top panel) from *Leukemia* to other cancers, here *Lung cancer* and *Non-Hodgkin lymphoma*.

In the reduced network of cancer, Fig 6 (top panel), we connect to each cancer types the two preferentially linked countries, i.e., for each cancer type *cr*, the two countries *cn*_1_ and *cn*_2_ giving the two highest value ${\left(G_{rr}+G_{\text{qrnd}}\right)}_{cn,cr}$. We observe that cancers affecting digestive system point preferentially to Asian countries with the exception of Great Britain and Chile (*Liver cancer* points to Thailand and Saudi Arabia, *Stomach cancer* to Mongolia and Chile, *Colorectal cancer* to Philippines and Great Britain). This results are correlated to the fact that high mortality rates for *Liver cancer* are found in Asia (with the highest death rates for Eastern Asia [44]), and for *Stomach cancer* in Eastern Asia and South America [45, 46]. In the other hand *Colorectal cancer* epidemiology clearly states [47] that the highest incidence rates are found for Western countries such as Great Britain. The appearance of Philippines pointed by *Colorectal cancer* is an artifact due to the mention in the corresponding 2017 Wikipedia article of Corazon Aquino, former president of the Philippines who was diagnosed with this cancer type. Blood cancer types points preferentially to African countries with the exception of Cambodia pointed by *Hodgkin lymphoma*. At first sight this results can appear surprising since these blood cancers are found worldwide with incidence rates highest for Western countries and lowest for African countries [48]. In fact there is a Non-Hodgkin lymphoma, the Burkitt’s lymphoma [49], which mainly affects children in malaria endemic region, i.e., Equatorial and Sub-Equatorial Africa and Eastern Asia. Countries pointed by blood cancer types, i.e., Liberia, Zambia, Cameroon, Gabon and Cambodia, belong to these regions. Let us note that these cancers and countries are connected through hidden links. *Melanoma* points to Australia, which is, with New Zealand [50], the country having the highest rate of *Melanoma*, and points to Peru, where nine 2400 years old mummies have been found with apparent signs of *Melanoma* [50]. *Prostate cancer* points preferentially to Japan, due to its exceptional low incidence on Japanese population in Japan and abroad [51, 52], to Nigeria, since it is believe that black population is particularly at risk [53]. *Lung cancer* points to Germany, where in 1929 it was shown for the first time a correlation between smoking and *Lung cancer* [54, 55], and to Bhutan which adopted a complete smoking ban since 2005 [54]. Hidden link from *Breast cancer* to Republic of San Marino should be related to the fact that inhabitants of San Marino commemorate Saint Agatha, patroness of the Republic and of breast cancer patients [56]. Hidden link from *Breast cancer* to Cambodia is more difficult to interpret.

Let us now consider the reduced Google matrix associated to *N*_*r*_ = *N*_*cr*_ + *N*_*d*_ = 240 May 2017 English Wikipedia articles devoted to *N*_*cr*_ = 37 cancer types and to *N*_*d*_ = 203 cancer drugs. As above the reduced network of cancer can be constructed (Fig 6, bottom panel). The construction process ends at the 4th iteration. The main structure of reduced network of cancers is the same as the previous with some exceptions. *Pancreatic cancer* is added to the digestive system cancers cluster and via hidden links, *Melanoma* points now to *Skin cancer* which points to *Breast cancer*. Consequently we observe a new cluster of thoracic region cancers comprising *Skin, Breast, Lung cancers* and *Melanoma*. Let us connect to each cancer types the two preferentially linked cancer drugs, i.e., for each cancer type *cr*, the two cancer drugs *d*_1_ and *d*_2_ giving the two highest value ${\left(G_{rr}+G_{\text{qrnd}}\right)}_{d,cr}$. Using DrugBank database [29], we easily check that indeed each drug is currently used to treat the cancer type to which it is connected. Also, closely connected cancer types share the same medication, e.g., *Skin cancer* and *Melanoma* are treated by *Vemurafenib* and *Dabrafenib* which are enzyme inhibitor of BRAF gene [57], *Leukemia* and *Non-Hodgkin lymphoma* are treated by the antibody *Rituximab* targeting B-lymphocyte antigen CD20 [58]. On the other hand non connected cancer types can in some cases share the same medication, the monoclonal antibody *Trastuzumab* typically used for *Breast cancer* is now also considered as a drug for *Stomach cancer* since these two cancer types overexpress the HER2 gene [59]. Let us note that hidden links connecting *Non-Hodgkin lymphoma* to *Cyclophosphamide* and *Rituximab* capture also a current medication reported in DrugBank database [29].

The reduced network of cancers shown in Fig 6 depict in a relevant manner interactions between cancers, cancer-country and cancer-drug interactions through Wikipedia.

### World countries sensitivity to cancers

We consider the reduced Google matrix associated to the set of *N*_*r*_ = *N*_*cr*_ + *N*_*cn*_ = 232 Wikipedia articles constituted of *N*_*cr*_ = 37 articles devoted to cancer types and of *N*_*cn*_ = 195 articles devoted to countries. We compute the PageRank sensitivity *D*(*cr* → *cn*,*cn*), i.e., the infinitesimal rate of variation of PageRank probability *P*(*cn*) when the directed link *cr* → *cn*, ${\left(G_{R}\right)}_{cn,cr}$, is increased by an amount $\delta {\left(G_{R}\right)}_{cn,cr}$, where *δ* is an infinitesimal.

**Fig 7:**
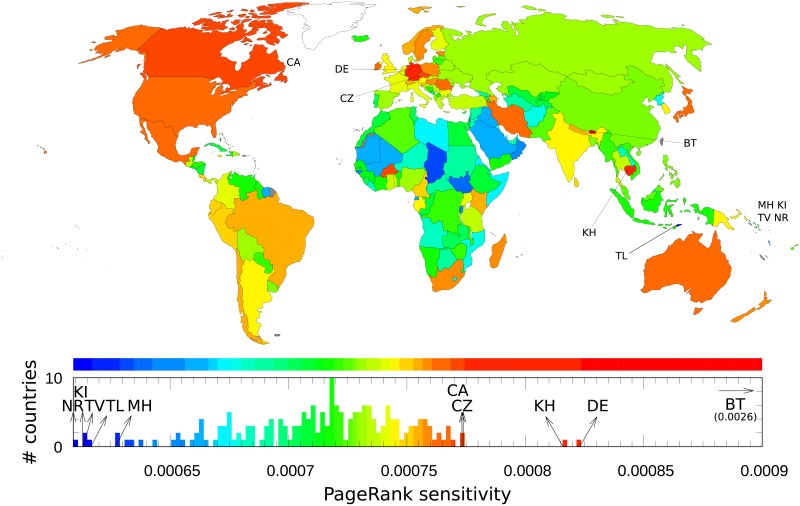
Sensitivity of countries to *Lung cancer*. A country *cn* is colored according to its diagonal PageRank sensitivity *D*(*cr* → *cn*,*cn*) to *Lung cancer*.

Fig 7 shows the world distribution of PageRank sensitivity *D*(*cr* → *cn*,*cn*) to *Lung cancer*. In Fig 7, color categories are obtained using the Jenks natural breaks classification method [60]. The most sensitive countries are, as discussed in the previous section, Bhutan and Germany mainly because these countries are directly cited in Wikipedia’s *Lung cancer* article. Besides articles devoted to these two countries the others are not directly linked from the *Lung cancer* article and the results obtained in Fig 7 (top panel) is consistent with GLOBOCAN 2018 data [4]: apart Micronesia/Polynesia, the most affected countries, in term of incidence rates, are Eastern Europe, Eastern Asia, Western Europe, and, Southern Europe for males, and, Northern America, Northern Europe, Western Europe, and, Australia/New Zealand for females. The less affected are African countries for both sexes. Let us note that although incidence rates are very high for males in Micronesia/Polynesia according to [4], this fact is not captured by Wikipedia since Nauru, Kiribati, Tuvalu, Marshall Islands are the less PageRank sensitive countries. This is certainly due to the fact that articles devoted to these sovereign states are among the worst ranked articles devoted to countries in the May 2017 English Wikipedia ranking using PageRank algorithm. Their respective ranks are Nauru *K* = 7085, Kiribati *K* = 7659, Tuvalu *K* = 6201, Marshall Islands *K* = 4549 to compare e.g. with USA *K* = 1, France *K* = 4, Germany *K* = 5, etc (see PageRank indices of countries in [28]).

As complementary information, sensitivities of countries to *Breast cancer* and to *Leukemia* are given in [28].

**Fig 8:**
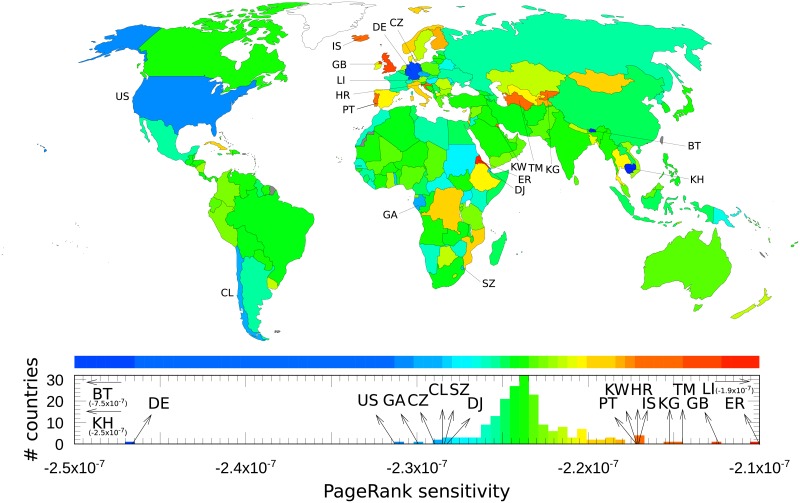
Sensitivity of countries to cancer → drug link variation. A country *cn* is colored according to its nondiagonal PageRank sensitivity *D*(*cr* → *d*,*cn*) to *cr* → *d* link variation. Variation of *Lung cancer* → *Bevacizumab* link is considered.

In order to investigate cancer—drug interactions it is also possible to represent sensitivity of countries to the variation of links from a cancer to a drug. As an illustration, Fig 8 shows countries PageRank sensitivities to variation of *Lung cancer* → *Bevacizumab* link. We see that in this case the sensitivity of countries is significantly reduced comparing to the direct sensitivity influence of lung cancer on world countries shown in Fig 7. Since the influence of this link variation is indirect for countries it is rather difficult to recover due to what indirect links the influence for specific countries is bigger or smaller. Among the most affected European countries we find Lichtenstein, Great Britain, Iceland, Portugal and Croatia while Germany and the Czech Republic are mostly unaffected. Another example of sensitivity of countries to cancer-drug link variation is given in [28].

### Interactions between cancers and drugs

Let us investigate interactions between cancers and drugs considering the subnetwork of *N*_*cr*_ = 37 cancers (see Table 1) and of the first 37 cancer drugs appearing in the PageRank ordered list Table 3. We do not consider *Talc* here since it is widely used in not only pharmaceutical industries.

**Fig 9:**
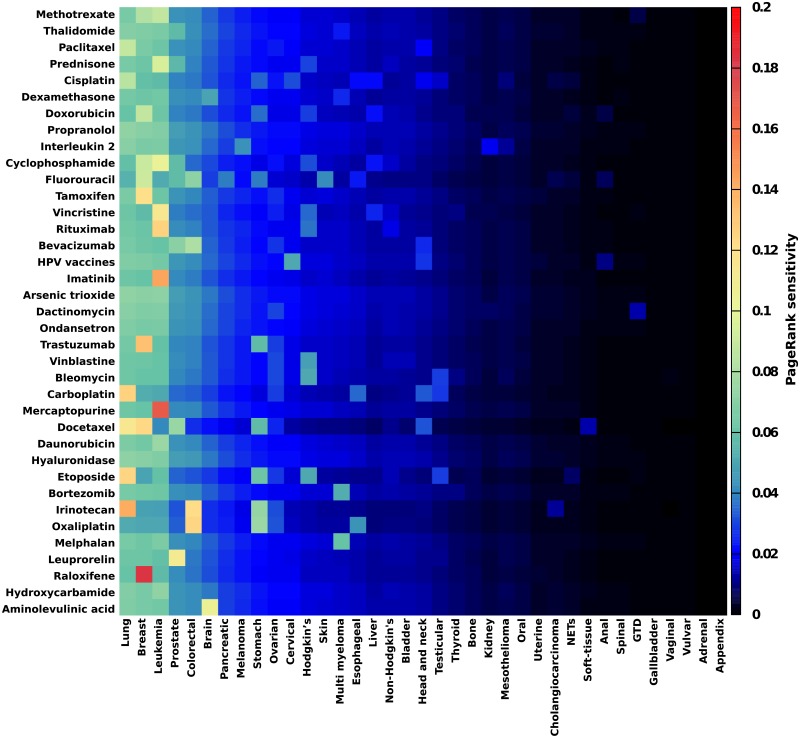
Sensitivity of cancers to drugs. The PageRank sensitivity *D*(*cr* → *d*,*cr*) of cancers to cancer drugs is represented. Here we consider the first 37 cancers (*cr*) listed in Table 3 and the first 37 drugs (*d*) listed in Table 2 (*Talc* has been removed as its article is too general).

We consider the sensitivity of cancer to drugs via the computation of *D*(*cr* → *d*,*cr*) presented in Fig 9. Although the PageRank sensitivity is computed using the logarithmic derivative of the PageRank, globally the most sensitives cancers are the ones with the highest PageRank probability, i.e., the ones with lowest PageRank indices *K* (see Fig 2 and Table 3): *Lung cancer* is mostly sensitive to *Irinotecan*, *Etoposide*, *Carboplatin*, *Breast cancer* to *Raloxifene*, *Trastuzumab*, *Docetaxel*, *Leukemia* to *Mercaptopurine*, *Imatinib*, *Rituximab*, etc. Following the National Cancer Institute [25] and/or DrugBank [29] databases, these associations cancer—drug are indeed approved.

**Fig 10:**
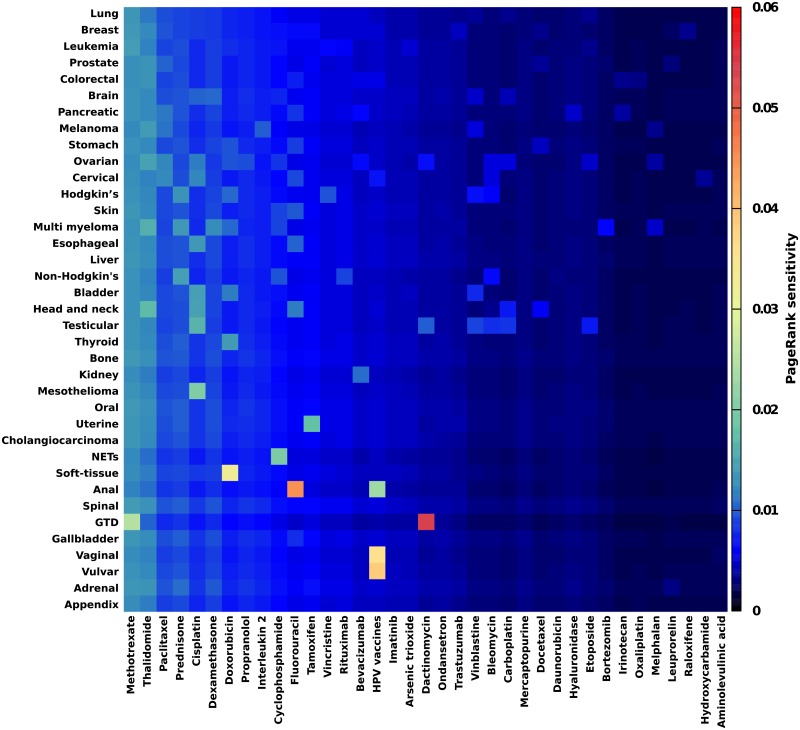
Sensitivity of drugs to cancers. The PageRank sensitivity *D*(*d* → *cr*,*d*) of cancer drugs to cancers is represented. Here we consider the first 37 cancers (*cr*) listed in Table 3 and the first 37 drugs (*d*) listed in Table 2 (*Talc* has been removed as its article is too general).

Fig 10 shows the complementary view of the sensitivity of drugs to cancers obtained from the computation of *D*(*d* → *cr*,*d*). Here the most sensitive drugs are *Dactinomycin* to *Gestational trophoblastic disease*, *HPV vaccines* to *Vulvar* and *Vaginal cancers*, *Fluorouracil* to *Anal cancer*, *Doxorubicin* to *Soft-tissue cancers*, etc. Again the National Cancer Institute [25] and DrugBank [29] databases report these possible drug—cancer associations.

Let us consider directly the reduced Google matrix associated to the top 20 cancer types and top 20 cancer drugs according to May 2017 English Wikipedia PageRank list (Table 3). This reduced Google matrix *G*_R_ and its *G*_*rr*_, *G*_pr_ and *G*_qrnd_ components are shown in Fig 5.

For each cancer *cr* of the 20 most influent cancer types in May 2017 English Wikipedia let us determine the three most connected drugs *d*, i.e., the three drugs with the highest value of ${\left(G_{rr}+G_{\text{qrnd}}\right)}_{d,cr}$. In Table 6 we show the May 2017 English Wikipedia prescription for each one of the top 20 cancer types. Most of the prescribed drugs are approved drugs for the considered cancer types according to National Cancer Institute [25] and DrugBank [29]. Some of the Wikipedia proposed drugs are in fact subject of passed, ongoing or planned clinical trials. Only Dexamethasone is in fact not specific to *Brain tumor* since it is a corticosteroid used to treat inflammation in many medical conditions [61]. We observe that hidden links gives also accurate medication, see drugs associated to *Non-Hodgkin lymphoma* and *Bladder cancer* in Table 6.

**Table 6:** Drug prescription by Wikipedia for the top 20 most influential cancer types and comparison with prescriptions by National Cancer Institute and DrugBank. For each of the top 20 cancer types ranked in May 2017 English Wikipedia using PageRank algorithm (see Table 3), we give the three strongest cancer → drug links, i.e., for a given cancer type *cr* we select the three cancer drugs *d* with the highest values ${\left(G_{rr}+G_{\text{qr}}\right)}_{d,cr}$. Drug with red background indicates a pure hidden cancer → drug link, i.e., the cancer type article in Wikipedia does not refer directly to the drug. For each cancer → drug link, the drug is followed by a ✔ mark if it is indeed prescribed for the cancer type according to National Cancer Institute [25] and/or DrugBank [29]; by a ▲ mark if the drug appears only as a subject of passed, ongoing or planned clinical trials reported for the cancer type in DrugBank; and by a ✘ mark otherwise.

	Cancer	1st drug		2nd drug		3rd drug	
1	Lung cancer	Erlotinib	✔	Crizotinib	✔	Cisplatin	✔
2	Breast cancer	Tamoxifen	✔	Trastuzumab	✔	Methotrexate	✔
3	Leukemia	Imatinib	✔	Rituximab	✔	Methotrexate	✔
4	Prostate cancer	Enzalutamide	✔	Cyclophosphamide	▲	Prednisone	✔
5	Colorectal cancer	Fluorouracil	✔	Irinotecan	✔	Bevacizumab	✔
6	Brain tumor	Temozolomide	✔	Dexamethasone	✘^a^	Aminolevulinic acid	▲
7	Pancreatic cancer	Fluorouracil	✔	Gemcitabine	✔	Protein-bound paclitaxel	✔
8	Melanoma	Vemurafenib	✔	Dabrafenib	✔	Trametinib	✔
9	Stomach cancer	Trastuzumab	✔	Doxorubicin	✔	Cisplatin	▲
10	Ovarian cancer	Cisplatin	✔	Tamoxifen	▲	Bevacizumab	✔
11	Cervical cancer	HPV vaccines	✔	Cisplatin	✔	Topotecan	✔
12	Hodgkin’s lymphoma	Prednisone	✔	Cyclophosphamide	✔	Vincristine	✔
13	Skin cancer	Vemurafenib	✔	Dabrafenib	✔	Fluorouracil	✔
14	Multiple myeloma	Dexamethasone	▲	Elotuzumab	✔	Bortezomib	✔
15	Esophageal cancer	Cisplatin	✔	Carboplatin	✔	Fluorouracil	✔
16	Liver cancer	Doxorubicin	▲	Cisplatin	▲	Sorafenib	✔
17	Non-Hodgkin’s lymphoma	Cyclophosphamide	✔	Rituximab	✔	Prednisone	✔
18	Bladder cancer	Doxorubicin	✔	Cisplatin	✔	Methotrexate	✔
19	Head and neck cancer	Cetuximab	✔	Paclitaxel	✔	Cisplatin	✔
20	Testicular cancer	Etoposide	✔	Cisplatin	✔	Bleomycin	✔

Notes:

^a^ Dexamethasone may be used to decrease swelling around the tumor.

Conversely for each cancer drug *d* of the 20 most influent cancer drugs in 2007 English Wikipedia we determine the three most connected cancer types *cr*, i.e., the three cancer types with the highest value of ${\left(G_{rr}+G_{\text{qrnd}}\right)}_{cr,d}$. In Table 7 we show for which cancers a drug is prescribed according to May 2017 English Wikipedia. Again the results are globally in accordance with National Cancer Institute [25] and DrugBank [29] databases. We note that hidden links here correspond mainly to clinical trials, e.g., Imatinib is an approved drug for treatment of certain forms of *Leukemia*, but experiments were or will be done for *Breast cancer* and *Prostate cancer*.

**Table 7:** According to Wikipedia for which cancer type is prescribed the top 20 most influential cancer drugs and comparison with prescriptions by National Cancer Institute and DrugBank. For each of the top 20 cancer drugs ranked in May 2017 English Wikipedia using PageRank algorithm (see Table 3), we give the three strongest drug → cancer links, i.e., for a given drug *d* we select the three cancer types *cr* with the highest values ${\left(G_{rr}+G_{\text{qr}}\right)}_{cr,d}$. Cancer type with red background indicates a pure hidden drug → cancer link, i.e., the drug article in Wikipedia does not refer directly to the cancer type. For each drug → cancer link, the cancer type is followed by a ✔ mark if the drug is indeed prescribed for the cancer type according to National Cancer Institute [25] and/or DrugBank [29]; by a ▲ mark if the drug appears only as a subject of passed, ongoing or planned clinical trials reported for the cancer type in DrugBank; and by a ✘ mark otherwise.

	Drug	1st cancer type		2nd cancer type		3rd cancer type	
1	Talc	Ovarian cancer	✘	Lung cancer	✘	Breast cancer	✘
2	Methotrexate	Leukemia	✔	Breast cancer	✔	Lung cancer	✔
3	Thalidomide	Multiple myeloma	✔	Breast cancer	▲	Prostate cancer	▲
4	Paclitaxel	Breast cancer	✔	Lung cancer	✔	Ovarian cancer	✔
5	Prednisone	Multiple myeloma	▲	Non-Hodgkin lymphoma	✔	Hodgkin’s lymphoma	✔
6	Cisplatin	Lung cancer	✔	Testicular cancer	✔	Breast cancer	✔
7	Dexamethasone	Multiple myeloma	▲	Brain tumor	✘^a^	Leukemia	✔
8	Doxorubicin	Leukemia	✔	Hodgkin’s lymphoma	✔	Breast cancer	✔
9	Propranolol	Ovarian cancer	▲	Brain tumor	✘	Colorectal cancer	▲
10	Interleukin 2	Melanoma	✔	Leukemia	▲	Hodgkin’s lymphoma	▲
11	Cyclophosphamide	Leukemia	✔	Multiple myeloma	✔	Breast cancer	✔
12	Fluorouracil	Colorectal cancer	✔	Breast cancer	✔	Stomach cancer	✔
13	Tamoxifen	Breast cancer	✔	Uterine cancer	▲	Prostate cancer	▲
14	Vincristine	Leukemia	✔	Hodgkin’s lymphoma	✔	Lung cancer	✔
15	Rituximab	Leukemia	✔	Non-Hodgkin lymphoma	✔	Multiple myeloma	▲
16	Bevacizumab	Breast cancer	✔	Colorectal cancer	✔	Lung cancer	✔
17	HPV vaccines	Cervical cancer	✔	Breast cancer	✘	Colorectal cancer	✘
18	Imatinib	Leukemia	✔	Breast cancer	▲	Prostate cancer	▲
19	Arsenic trioxide	Leukemia	✔	Brain tumor	▲	Breast cancer	▲
20	Dactinomycin	GTD^b^	✔	Testicular cancer	✔	Ovarian cancer	✔

Notes:

^a^ Dexamethasone may be used to decrease swelling around the tumor.

^b^ Gestational trophoblastic disease.

It would be interesting to thoroughly study the most connected drugs and cancer types from the hidden contributions only, i.e., from ${\left(G_{\text{qrnd}}\right)}_{cr,d\;\text{or}\;d,cr}$ matrix elements only, in order to test the possible predictive power of the reduced Google matrix analysis of Wikipedia networks. This study is beyond the scope of the present work and will be considered in a subsequent one.

## Conclusion

Using PageRank and CheiRank algorithms, we investigate global influences of 37 cancer types and 203 cancer drugs through the prism of Human knowledge encoded in the English edition of Wikipedia considered as a complex network. From the ranking of Wikipedia articles using PageRank algorithm we extract the ranking of the most influent cancers according to Wikipedia. This ranking is in good agreement with rankings, by either mortality rates or yearly new cases, extracted from WHO GLOBOCAN 2018 [2] and Global Burden of Diseases study 2017 [5] databases.

The recently developed algorithm of the reduced Google matrix allows to construct a reduced network of cancers taking into account all the information aggregated in Wikipedia. This network exhibits direct and hidden links between the most influent cancers which form clusters of similar or related cancer types. The reduced Google matrix gives also countries or cancer drugs which are preferentially linked to the most influent cancers. Inferred relations between cancer types and countries obtained from Wikipedia network analysis are in accordance with global epidemiology literature. The PageRank sensitivity of countries to cancer types gives also a complementary tool corroborating epidemiological analysis. As far as we know, it is the first study highlighting correspondence between Wikipedia network analysis and disease burden or epidemiological studies. Inferred interactions between cancers and cancer drugs allows to determine drug prescriptions by Wikipedia for a specific cancer. These Wikipedia prescriptions appear to be compatible with approved medications reported in National Cancer Institute [25] and DrugBank [29] databases.

The reduced Google matrix algorithm allows to determine a clear and compact description of global influences and interactions of cancer types and cancer drugs integrating well documented medical aspects but also historical, and societal aspects, all encoded in the huge amount of knowledge aggregated in Wikipedia since 2001.
